# Avoiding Unnecessary Repeat Laboratory Testing

**DOI:** 10.7759/cureus.5872

**Published:** 2019-10-09

**Authors:** Tarang Patel, Ethan Karle, Armin Krvavac

**Affiliations:** 1 Internal Medicine, University of Missouri Healthcare, Columbia, USA; 2 Pulmonary & Critical Care, University of Missouri Healthcare, Columbia, USA

**Keywords:** high value care, surreptitious use of insulin, cost-effective, factitious disorder, laboratory testing

## Abstract

We present a 44-year-old Caucasian female with a history of diabetes mellitus admitted to the intensive care unit (ICU) for refractory hypoglycemia with an initial blood glucose of 39 mg/dl. The initial evaluation included a random insulin level, C-peptide, Hemoglobin A1c, and a sulfonylurea screen that were ordered when the patient's blood sugar was 39 mg/dL. She was discharged after demonstrating euglycemia. The test results for sulfonylurea screen, insulin, and C-peptide levels were obtained one day after discharge. The insulin level was elevated, and C-peptide was inappropriately low, establishing the diagnosis of surreptitious exogenous insulin use. Four days after discharge, the patient was readmitted to the same ICU with a similar presentation of refractory hypoglycemia. Once again, the sulfonylurea screen, along with the insulin and C-peptide levels were ordered as there was no mention of the previously obtained results in the discharge summary. The discrepancy between random insulin and C-peptide levels reaffirmed the diagnosis of surreptitious exogenous use of insulin. As high-value medical care becomes a focal point in medicine, the costs, root causes, and impacts of inappropriate laboratory testing must be understood. Upwards of 25% of ordered laboratory tests are unnecessary. Physicians' failure to follow-up on results of correctly ordered tests and repeat testing despite established diagnosis is a significant cause of unneeded laboratory testing. Best practice guidelines recommend a reduction in unnecessary laboratory testing by implementing computer-based solutions to maximize the identification of duplicate requests and to promote clinical education at the time of laboratory test ordering.

## Introduction

Medical care over the past decade has transitioned to emphasize on the provider's understanding of high-value care. An area of focus regarding high-value care is to minimize cost by placing emphasis on minimizing inappropriate laboratory testing. This approach puts the responsibility of assessing the need for laboratory testing on physicians and promotes the utilization of electronic safeguards to help achieve this goal. Major barriers to minimizing inappropriate laboratory testing predominately stem as a result of a combination of provider failure to follow-up previous results, the practice of defensive medicine, and inappropriate protocol-based laboratory testing. It is imperative that as providers, we be aware of the existing barriers to minimizing inappropriate laboratory testing. 

## Case presentation

A 44-year-old Caucasian female with a history of diabetes mellitus (DM), depression, and posttraumatic stress disorder was admitted to the medical intensive care unit (ICU) for refractory hypoglycemia. The patient reported nausea, vomiting, and inability to tolerate oral intake. Her initial blood glucose was 39 mg/dl and only improved after escalating the infusion of intravenous 10% dextrose (D10) to 200 mL/h. Patient-reported diagnosis of DM type II in 2011 that was treated with metformin and insulin. Three years later, she successfully weaned off of insulin therapy after improvement in her glycemic control. She reported appropriately discarding all of her insulin by taking it to the police station. Furthermore, she denied having access to insulin, sulfonylureas, or other oral antidiabetic medications.

**Figure 1:**
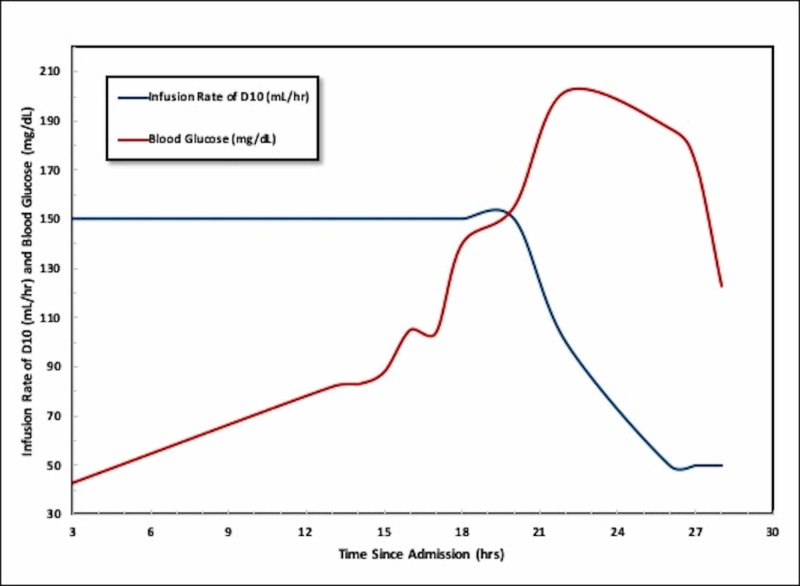
Graphical Representation of the patient’s blood glucose in mg/dL (Red) and rate of intravenous D10 in mL/hr (Blue) administered plotted over time, represented as hours since admission

The differential diagnosis for refractory hypoglycemia, in this case, included: hepatic dysfunction, nutritional deficiencies, diminished oral intake, insulinoma, and surreptitious exogenous administration of insulin or sulfonylurea. The initial evaluation included a random insulin level, C-peptide, Hemoglobin A1c, and a sulfonylurea screen that were obtained when the patient's blood sugar was 39 mg/dL. The hemoglobin A1c was 5.1%. The patient's blood sugar spontaneously improved; allowing her to be weaned off D10 infusion (Figure 1). She was discharged after demonstrating normal blood sugars consistently and tolerating oral intake.

The results of sulfonylurea-screen, along with the insulin and C-peptide levels, were obtained one day after discharge. The insulin level was elevated at 216.43 mcunit/ml, C-peptide was inappropriately low at 1.619 nmol/L, and the sulfonylurea screening test was negative. Given the constellation of presenting symptoms as well as the elevated insulin level with a low C-peptide, the diagnosis of surreptitious exogenous insulin use was confirmed [1].

Four days after discharge, the patient was readmitted to the same ICU with a similar presentation of refractory hypoglycemia. She once again required intravenous D10 to maintain euglycemia. Sulfonylurea screen, along with the insulin and C-peptide levels, were ordered again as there was no mention of the previous laboratory studies in the discharge summary. The random insulin level was elevated, and the C-peptide level was low (Table 1). The discrepancy between random insulin and C-peptide levels reaffirmed the diagnosis of surreptitious exogenous use of insulin. Psychiatry was consulted and established the diagnosis of factitious disorder. The patient vehemently continued to deny surreptitious, exogenous use of insulin. She was ultimately discharged with close psychiatric and primary care follow-up.

**Table 1:** Comparing the results of laboratory testing between the patient’s initial admission and the patient’s re-admission four days after discharge

Evaluation of Refractory Hypoglycemia: Review of Admission Labs		
Test Name	Admission One	Admission Two
Insulin Level (mcunit/mL)	216.43	90.26
C-Peptide (nmol/L)	1.619	0.084
Hemoglobin A1c (%)	5.1	Not Applicable
Sulfonylurea Screening Test	Negative	Negative

## Discussion

The evaluation of hypoglycemia requires clinicians to be aware of the broad differential diagnoses that exist, the appropriate diagnostic work-up, and the interpretation of the diagnostic testing. The cornerstone of identifying patients in whom to pursue evaluation for hypoglycemia requires satisfying Whipple’s Triad (symptoms consistent with hypoglycemia, verification of a low plasma glucose level using a precise method, and resolution of symptoms with the raising of plasma glucose levels) [2]. Extensive testing to elucidate the etiology of hypoglycemia is recommended only for patients with symptomatic hypoglycemia, more specifically, for those in whom conditions of Whipple’s Triad are met. Causes of symptomatic hypoglycemia include sepsis, insulinoma, insulin autoimmune hypoglycemia, as well as the accidental, surreptitious, or malicious use of sulfonylurea drugs or insulin products [2]. The diagnostic work-up largely revolves around laboratory testing, which should be obtained at the time of the hypoglycemic event. This laboratory testing should include serum glucose levels, insulin levels, C-peptide levels, and screening for sulfonylurea drugs [2]. 

The interplay between insulin levels, C-peptide levels, and the sulfonylurea drug screen will aid in making the correct diagnosis. Elevated insulin levels (greater than 3 mcunit/ml) can be the result of exogenous use of insulin or an insulinoma. In these cases, the naturally occurring C-peptide (a byproduct of endogenously produced insulin) can be used to distinguish the two diagnoses. C-peptide levels are low or normal in cases of exogenous insulin use high if hypoglycemia is the result of an insulinoma. Both exogenous use of insulin and insulinoma, require a negative sulfonylurea drug screen to confirm the diagnosis. Accidental, surreptitious, or malicious use of sulfonylureas mimics the laboratory findings seen in an insulinoma, with the exception being the positive sulfonylurea drug screen. Symptomatic hypoglycemia is a complex diagnosis with a broad differential, which necessitates providers to be aware of the appropriate evaluation and correct interpretation of various laboratory studies. 

As high-value medical care becomes a focal point in medicine, the costs, root causes, and impacts of inappropriate laboratory testing must be understood. Upwards of 25% of ordered laboratory tests are unnecessary [3]. This problem is magnified in the ICU, where laboratory testing can represent up to 10% of the total cost of hospitalization [4]. Additionally, a 2005 article identified defensive medicine as a source of unnecessary laboratory testing. Of those surveyed, 82% reported practicing defensive medicine, and 59% reported ordering medically unnecessary tests [5]. Protocol-based laboratory requesting and a lack of physician awareness of recommended repeat testing intervals also contribute to inappropriate laboratory testing [3]. More importantly, physicians' failure to follow-up on results of correctly ordered tests and repeat testing despite established diagnosis remain major causes of unneeded laboratory testing [3].

In addition to a careful review of the medical chart and accurate clinical documentation, several interventions have been identified to curtail unnecessary laboratory testing. Healthcare providers in an ICU demonstrated a significant reduction in average daily laboratory testing when provided with an itemized list of patient charges for diagnostic tests [6]. Best practice guidelines recommend a reduction in unnecessary laboratory testing by implementing computer-based solutions to maximize the identification of duplicate requests and to promote clinical education at the time of laboratory test ordering [3].

## Conclusions

Unnecessary laboratory testing not only represents a financial burden on the healthcare system but also represents an unnecessary burden on the patient. Unwarranted testing can result in increased patient discomfort, anxiety, and improper diagnosis due to false-positive results; potentially leading to additional referrals, imaging, and laboratory testing.
